# Equine Chorionic Gonadotropin as an Effective FSH Replacement for In Vitro Ovine Follicle and Oocyte Development

**DOI:** 10.3390/ijms222212422

**Published:** 2021-11-17

**Authors:** Chiara Di Berardino, Alessia Peserico, Giulia Capacchietti, Martina Crociati, Maurizio Monaci, Umberto Tosi, Annunziata Mauro, Valentina Russo, Nicola Bernabò, Barbara Barboni

**Affiliations:** 1 Faculty of Bioscience and Technology for Food, Agriculture and Environment, University of Teramo, 64100 Teramo, Italy; cdiberardino@unite.it (C.D.B.); apeserico@unite.it (A.P.); gcapacchietti@unite.it (G.C.); utosi@unite.it (U.T.); amauro@unite.it (A.M.); vrusso@unite.it (V.R.); nbernabo@unite.it (N.B.); 2 Department of Veterinary Medicine, University of Perugia, 06126 Perugia, Italy; martina.crociati@unipg.it (M.C.); maurizio.monaci@unipg.it (M.M.); 3 Centre for Perinatal and Reproductive Medicine, University of Perugia, 06126 Perugia, Italy; 4 Institute of Biochemistry and Cell Biology (IBBC), National Research Council, A. Buzzati-Traverso Campus, via E. Ramarini 32, Monterotondo Scalo, I-00015 Rome, Italy

**Keywords:** in vitro follicle culture, follicle growth, eCG, FSH, steroidogenesis, oocyte meiotic competence, ovine

## Abstract

The use of assisted reproductive technologies (ART) still requires strategies through which to maximize individual fertility chances. In vitro folliculogenesis (*iv*F) may represent a valid option to convey the large source of immature oocytes in ART. Several efforts have been made to set up *iv*F cultural protocols in medium-sized mammals, starting with the identification of the most suitable gonadotropic stimulus. In this study, Equine Chorionic Gonadotropin (eCG) is proposed as an alternative to Follicle Stimulating Hormone (FSH) based on its long superovulation use, trans-species validation, long half-life, and low costs. The use of 3D *iv*F on single-ovine preantral (PA) follicles allowed us to compare the hormonal effects and to validate their influence under two different cultural conditions. The use of eCG helped to stimulate the in vitro growth of ovine PA follicles by maximizing its influence under FBS-free medium. Higher performance of follicular growth, antrum formation, steroidogenic activity and gap junction marker expression were recorded. In addition, eCG, promoted a positive effect on the germinal compartment, leading to a higher incidence of meiotic competent oocytes. These findings should help to widen the use of eCG to *iv*F as a valid and largely available hormonal support enabling a synchronized in vitro follicle and oocyte development.

## 1. Introduction

Assisted reproductive technologies (ART) aim to overcome human infertility and to optimize reproductive exploitation in domestic animals and the preservation in endangered species.

In a female context, most of ART’s success has been through the optimization of the use of fully-grown competent oocytes to generate high quality embryos to be transferred. This is contrasted by the fact that most individual reproductive resources are enclosed into primordial and growing oocytes. These female gamete reserves, constituting the largest proportion of the total follicular population in the ovary [1,2], cannot be enrolled in ART, even though it has been demonstrated that their biological potential is maintained in cryopreserved ovarian tissues of several mammals (in sheep, goats and humans [3,4,5]).

One challenging biotechnological perspective is to mimic the process of follicle growth in vitro to support the acquisition of gamete meiotic and developmental competences, thus enlarging the availability on an individual basis of oocytes that can be enrolled with success in in vitro Maturation (IVM)/in vitro Fertilization (IVF) and embryo cultures. The *iv*F approach, which is of great value in valorizing large sources of immature gametes, represents a breakthrough in the reproduction field for therapeutic, experimental, and diagnostic purposes. So far, the *iv*F system has been developed successfully in mice [6,7] but it remains to be translated with efficiency to medium/large mammals. Currently, this limit is due to the longer period required for follicle/oocyte growth, the greater dimension of antral follicles enclosing competent oocytes and the difficulty in mimicking the environmentally favorable conditions that guarantee a synergic oocyte and somatic compartment development by preserving the tissue architecture [8,9,10,11,12,13,14,15,16,17]. To translate the in vitro culture systems to non-rodent models, a better comprehension of the physiology mechanisms triggering follicle growth initiation, sustaining the interplay between germinal and somatic compartment, and supporting the basic metabolic needs of early growing follicles, are required.

For this reason, even if primordial follicles represent the ideal starting source of gametes for *iv*F [1,2], most attention has been focused on medium/large mammals for the validation of the protocols supporting the growth of preantral (PA) follicles in order to reduce the cultural period required to complete the phase of oocyte growth [3,8,9,12,13,14,18,19].

The in vitro growth of isolated PA follicles [17,20,21,22,23], as well as the in vitro PA growth of follicles enclosed in strips of ovarian cortex [16,24,25], particular by culturing isolated PA follicles under two-dimensional (2D) and three-dimensional (3D) systems, has been successfully developed in non-rodent mammals and humans by several research groups.

Notably, the small ruminant model plays a pivotal role in this research context [12,24,26,27,28,29,30,31]. This is mainly due to its high translational value. Indeed, profound similarities with human reproductive physiology have been recognized through the small ruminant model: they are medium-sized mammals, mainly mono-ovulatory species; and the female gamete achieves evolutionistic growth during its transition from PA to early antral (EA) follicle [32,33,34]. In addition, researchers enjoy access to a substantial number of biological samples using the small ruminant model since the ovaries of small ruminants, even prepubertal animals, can be easily collected from slaughterhouses as discarded tissues. The high translational value of the medium-sized ruminant model is emphasized even further when using prepubertal ovaries, which exactly mimic the physiological structure of girls and young women [17,35].

Studies that have focused on the in vitro development of PA follicles in small ruminants have demonstrated the possibility of stimulating follicle growth in culture by inducing the activation of steroidogenesis and the secretion of follicle bioactive factors (Inhibin: sheep [23], goat [26,27]; or anti-Müllerian hormone [31]). However, several pieces of evidence, obtained by comparing the functional status of in vivo and in vitro developed follicles, showed that the current available protocols induced an earlier differentiation status, causing an increase in the rate of granulosa cell proliferation [31], the acceleration of follicular cell specialization [17], an earlier and faster acquisition of follicular maturation markers (*CYP19A1*, *FSHR*, *ESR2*, *INHA*, *INHBA*, *INHBB*, and *FST*), and the upregulation of theca-related genes (*LHR*, *CYP17A1*, and *CYP11A1*) [31].

The acceleration induced in follicle by the in vitro conditions may be responsible for the impaired follicles–oocyte dialogue affecting the in vitro oocyte growth process (buffalo [8], sheep [17], and goat [12]) and probably a precocious reduction in cumulus-oocyte metabolic coupling [17,31].

Cadoret et al. [31] demonstrated in sheep that the expression of oocyte-specific genes *ZP3*, *GJA4* (encoding the connexin 37 protein), *KIT*, and *BMP15* were significantly downregulated during culture.

However, despite the difference recorded between follicle growth in vivo and in vitro, enclosed oocytes may express growth capacity and a progressive degree of epigenetic maturation [17] associated with the acquisition of meiotic competence [3,17]. Sheep oocytes grown in vitro, indeed, were able to resume and complete meiosis, leading to meiotic competence that was equivalent to [17], and sometimes higher than, that of oocytes derived from early antral follicles developed in vivo [31,36].

In addition, despite the alterations recorded in oocyte gene expression [31], developmental competence has also been demonstrated in several ruminant models, such as bovine [37], buffalo [8], sheep [17], and goat [12] models.

The factors driving the PA to antral follicle transition are still poorly understood, but in most of in vitro systems, Follicle Stimulating Hormone (FSH) stimulation appeared to be crucial in inducing coordinated follicle and oocyte development. Recent studies used the responsiveness of the PA follicle gonadotropin alone or in combination with other growth factors [38,39,40,41,42]. Conversely, FSH in vivo seems to be a dispensable factor in follicles reaching the preantral antral stage and in the proliferation of granulosa cells, [2,43] and is controlled by intraovarian regulators [44], while follicles acquire FSH dependence during the transition from the preantral to the antral stage [45,46], where gonadotropin plays a pivotal role in regulating follicular fate [44]. Accordingly, a heavy requirement of FSH dependence was also reported in vitro in the stimulation growth from the PA stage to the antral stage [38,47].

In particular, FSH in small ruminants was recognized to induce in vitro PA follicle growth, antrum differentiation, steroidogenesis activation [17,31,48], the release of survival factors protecting the granulosa from apoptosis, and to stimulate the enhancement of gap junctional communication [4,23,49,50,51]. In addition, several pieces of evidence demonstrated that the in vitro influence of FSH on PA follicle growth is strictly dose-dependent.

While a low gonadotropin concentration seems to be required to promote oocyte growth [52], high concentrations of FSH enhance follicle development but have deleterious effects on the developmental capacity of oocytes [17,52]. Moreover, other factors seem to influence follicular performance, such as the use of sequential FSH concentration [30] and/or the different FSH origin [53].

However, besides the biological evidence obtained by different research groups regarding the effects of FSH, the debate continues.

For veterinary purposes, in order to induce the growth of follicles by ovaries and to increase the ovulation rate before the artificial insemination, Equine Chorionic Gonadotropin (eCG), previously named Pregnant Mare Serum Gonadotropin (PMSG), has been used successfully. Equine Chorionic Gonadotropin is a placental glycoprotein obtained from the serum of pregnant mares. It is widely used as a valid substitute for species-specific FSH in female mammals due to its longer circulatory half-life, its FSH like-activity, its easier use as a trans-species hormone, and its limited costs [54,55].

According to this premise, the research reported in this paper aimed to study the influence of two different gonadotropins (FSH vs. eCG) on in vitro follicle cultures of a single ovine PA follicle, collected at the large secondary stage. The *iv*F outcomes obtained by using the species-specific ovine FSH (oFSH) or the trans-species chorionic gonadotropin (eCG) were analyzed after 14 days of culture by comparing the FBS-supplemented medium with the FBS-free medium, follicle/oocyte in vivo/in vitro growth, the timing and percentage (%) of antrum differentiation, the results of an estradiol production assay, gene expression, as well as the percentage of Metaphase II (MII) oocytes after IVM.

## 2. Results

### 2.1. eCG Dose Effects on Follicular and Oocyte In Vitro Growth Cultured in FBS-Supplemented Medium

#### 2.1.1. Follicle Growth Parameters in FBS-Supplemented Medium

Before comparing the *iv*F influences of the two gonadotropins, eCG and oFSH, the most suitable concentration of chorionic gonadotropin was determined by designing an eCG dose-curve (0, 0.4, 4 and 40 IU/mL) and assessing the effect on follicle and oocyte maturation after 14 days of culture under previously validated conditions.

As summarized in Table 1, different concentrations of eCG impacted the follicle parameters. Specifically, the dose of 4 UI/mL increased the incidence of healthy grown follicles by improving the % of follicles reaching the EA stage (60% vs. approximately 30% of other doses). By contrast, healthy follicle growth was unaffected by the eCG concentration: the final diameters of the follicles treated with eCG 40, 4, 0.4 IU/mL were 337 ± 29 μm, 362 ± 50 μm, and 364.2 ± 44 μm, respectively. The mean follicle gradient growth induced by the eCG ranged around 40%, a value significantly greater than that recorded in the PA follicles incubated in the absence of gonadotropin stimulation (from 233.2 ± 3 to 256.2 ± 2 μm, *p* < 0.02).

**Figure 1:**
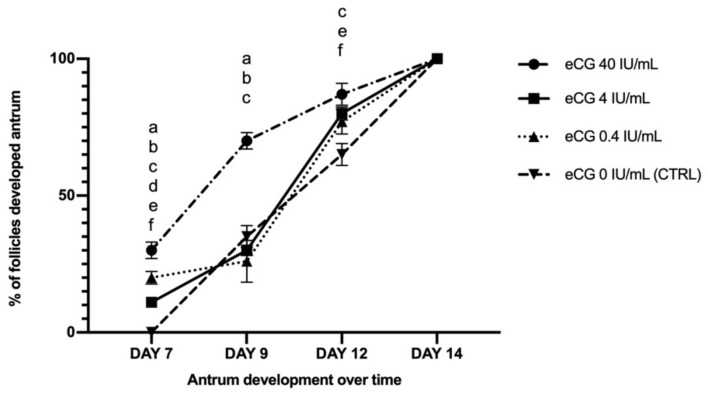
Development trend of the follicular antrum over time. Values with different superscripts are significantly different (*p* < 0.05). Specifically: ^a^ eCG 40 IU/mL vs. eCG 4 IU/mL; ^b^ eCG 40 IU/mL vs. eCG 0.4 IU/mL; ^c^ eCG 40 IU/mL vs. eCG 0 IU/mL (CTRL); ^d^ eCG 4 IU/mL vs. eCG 0.4 IU/mL; ^e^ eCG 4 IU/mL vs. eCG 0 IU/mL (CTRL); ^f^ eCG 0.4 IU/mL vs. eCG 0 IU/mL (CTRL). Three independent biological replicates were performed. For each biological replicate, a total of 30 follicles per group (eCG 40, 4, 0.4, 0 IU/mL) were cultured.

The analysis over time of antrum formation showed that eCG, independently of the doses, induced at day 12 the development of the EA stage in 80% of the follicles and 65% of the CTRL follicles (without eCG: *p* < 0.001). However, eCG 40 IU/mL induced a faster antrum formation, as summarized in Figure 1.

#### 2.1.2. Oocyte Meiotic Competence in FBS-Supplemented Medium

The higher percentage of healthy oocytes was collected from the follicles grown in vitro with 4 IU/mL of eCG (4 IU vs. 40 IU/mL: *p* < 0.0001; 4 IU vs. 0.4 IU/mL: *p* < 0.0002; 4 vs. 0 IU: *p* < 0.0001). The *iv*F performed with eCG promoted, independently of the dose, a significant increase in oocyte diameter (from approximately 74 μm to 103–106 μm). The oocyte size was similar to that recorded in the oocytes isolated from the in vivo EA follicles and significantly greater than those obtained under CTR conditions (absence of chorionic gonadotropin stimulation; diameter: 106 ± 3 μm; *p* < 0.05).

Notably, the meiotic competence reached by the oocytes obtained from the follicles grown in vitro was strictly eCG dose-dependent.

In more detail, the highest percentage of oocytes resuming meiosis (94.4%) was recorded in the 4 IU/mL eCG group. As summarized in Table 2, most of them (66.7%) were able to complete the meiotic cell cycle, reaching the Metaphase II (MII) stage. The percentage of GVBD and MII significantly decreased in the follicles cultured with lower concentrations of eCG (0 and 0.4 IU/mL) as well as in the 40 IU/mL group (see Table 2). The highest concentration of eCG did not enhance the meiotic competence of the oocytes derived from the in vitro cultured PA follicles (Table 2).

Based on the aforementioned data, eCG 4 IU/mL was selected as the more effective dose at inducing combined follicle and oocyte development under *iv*F conditions. Based on the follicle and oocyte performances, this dose was used in the subsequent experiments designed to compare the effect of eCG vs. oFSH (1 μg/mL) under *iv*F technique.

### 2.2. eCG and oFSH-Induced ivF Performance in PA Follicle Cultured in FBS-Supplemented Medium

#### 2.2.1. Follicle Growth Parameters Promoted in eCG and oFSH in FBS-Supplemented Medium

The comparison between eCG (4 IU/mL) and oFSH (1 μg/mL) was performed by assessing the *iv*F outcomes at the follicular and oocyte level.

**Figure 2:**
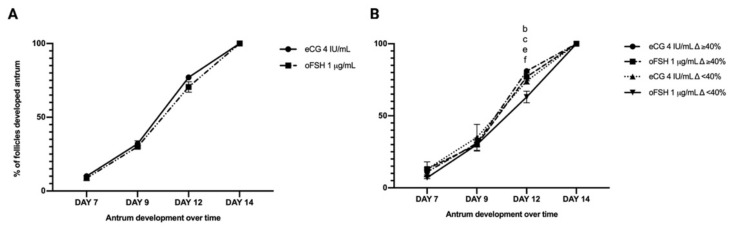
Development trend of the follicular antrum overtime. Antrum formation over time (**A**) and antrum formation overtime according to follicle ∆ growth % (**B**) following eCG and oFSH stimulation. Letters indicate significant differences between: eCG 4 IU/mL ∆ ≥ 40% vs. oFSH 1 ug/mL ∆ ≥ 40%; ^b^ eCG 4 IU/mL ∆ ≥ 40% vs. eCG 4 IU/mL ∆ < 40%; ^c^ eCG 4 IU/mL ∆ ≥ 40% vs. oFSH 1 ug/mL ∆ < 40%; ^e^ oFSH 1 ug/mL ∆ ≥ 40% vs. oFSH 1 ug/mL ∆ < 40%; ^f^ eCG 4 IU/mL ∆ < 40% vs. oFSH 1 ug/mL ∆ < 40%. Three independent biological replicates were performed. For each biological replicate, 66, 67, and 67 follicles per group (eCG 4 IU/mL and oFSH 1 μg/mL) were cultured.

No differences in *iv*F healthy follicles and the percentage of antrum formation were detected between the two gonadotropin treatments (Table 3 and Figure 2A).

However, analyzing the antrum formation over time in the two subpopulations of follicles classified on the basis of their Δ growth (class of follicles with a Δ growth higher or lower than the mean (< and ≥40%)) some differences between the gonadotropin-treated subpopulations were recorded. Indeed, eCG promoted a greater percentage of follicles with a Δ growth > 40% (81% vs. 60%: *p* < 0.0005; see Table 3). As reported in Figure 2B, the oFSH-treated follicles with a ∆ growth of less than 40% showed the smallest percentage of antrum development on Day 12 of *iv*F culture. Notably, a similar antrum differentiation trend was observed upon eCG treatment in both follicle subpopulations. (See Figure 2B)

#### 2.2.2. Oocyte Meiotic Competence Promoted in eCG and oFSH ivF Grown Oocytes

**Figure 3:**
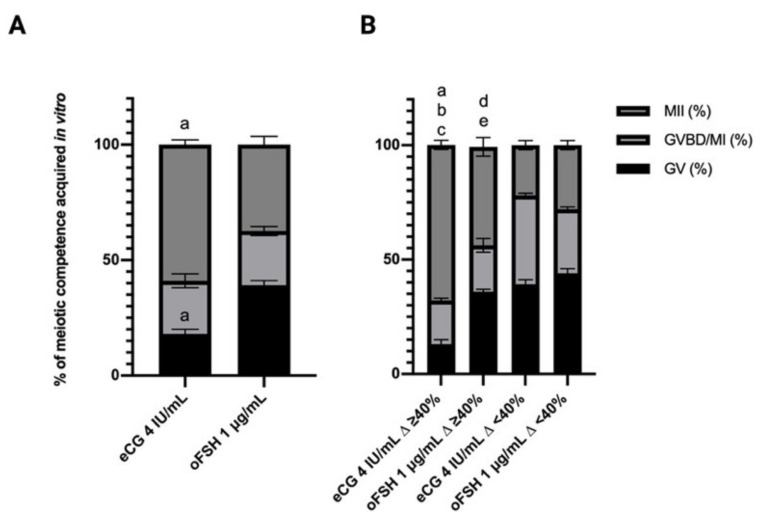
Percentage of oocyte meiotic competence acquired in vitro: an overview of germ cells’ nuclear stages. (**A**) All the oocytes recovered by follicular gonadotropin treatments independently of their ∆ growth%. (**B**) % of oocyte recovered from follicle with ∆ growth ≥ or < 40%. Letters indicate significant differences ^a^ eCG 4 IU ∆ ≥ 40% vs. FSH 1μg/mL ∆ ≥ 40%; ^b^ eCG 4 IU ∆ ≥ 40% vs. eCG 4 IU ∆ < 40%; ^c^ eCG 4 IU ∆ ≥ 40% vs. FSH 1μg/mL ∆ < 40%; ^d^ FSH 1μg/mL ∆ ≥ 40% vs. eCG 4 IU ∆ < 40%; ^e^ FSH 1μg/mL ∆ ≥ 40% vs. FSH 1μg/mL ∆ < 40%. MII: Metaphase II; GVBD/MI: Germinal Vesicle Break Down/Metaphase I; GV: Germinal Vesicle. Three independent biological replicates were performed with 66, 67, and 67 follicles per group (eCG 4 IU/mL and oFSH 1 μg/mL). For each *iv*F group (oFSH 1 μg/mL and eCG 4 IU/mL) 116 and 122 follicles, respectively, were analyzed at the end of the in vitro culture for both follicular and oocyte performance. Only those follicles that differentiated the follicular antrum, identified on the stereomicroscope as a transparent cavity filled with follicular fluid, were tested. For each *iv*F group (oFSH 1 μg/mL and eCG 4 IU/mL), 116 and 122 healthy oocytes were recovered and analyzed at the end of the in vitro culture to test the oocyte performance. Only the oocytes retrieved from follicles that differentiated the follicular antrum, identified on the stereomicroscope as a transparent cavity filled with follicular fluid, were tested.

As summarized in Table 4 and Figure 3A, the gonadotropin treatments did not influence the percentage of recovered healthy oocytes but, on the contrary, significantly affected the meiotic competence of in vitro grown oocytes (59% vs. 37.4% MII oocytes in eCG vs. oFSH groups, respectively *p* < 0.0008)

In addition, by analyzing the meiotic competences in the oocytes collected from the two subpopulations of EA follicles (∆ growth ≥ or <40%), a higher percentage of MII were observed in the oocytes collected from follicles with a ∆ growth >40%. In the eCG follicles, the completion of meiosis involved 68% vs. 22% of the oocytes collected from the follicles with growth ≥ or <40%, respectively. Similarly, the oFSH-derived oocytes reached the MII stage in 43% vs. 28% of oocyte collected from EA follicles with a ∆ growth ≥ or <40%, respectively (see Table 4 and Figure 3b).

In the light of the gathered data, eCG was the treatment that guaranteed the greatest acquisition of meiotic competence under *iv*F validated conditions, especially in those derived from follicles with a degree of growth higher than or equal to 40%.

### 2.3. Comparison between eCG and oFSH in Follicles Cultured in FBS-Free Medium

#### 2.3.1. PA Performances in eCG and oFSH In Vitro Grown PA Follicles Cultured in FBS-Free Medium

In order to evaluate whether the eCG’s positive influence on in vitro follicle culture was maintained under different cultural conditions, the oFSH and eCG stimulation was further assessed under in vitro follicle culture carried out in an FBS-free medium [56,57,58].

**Figure 4:**
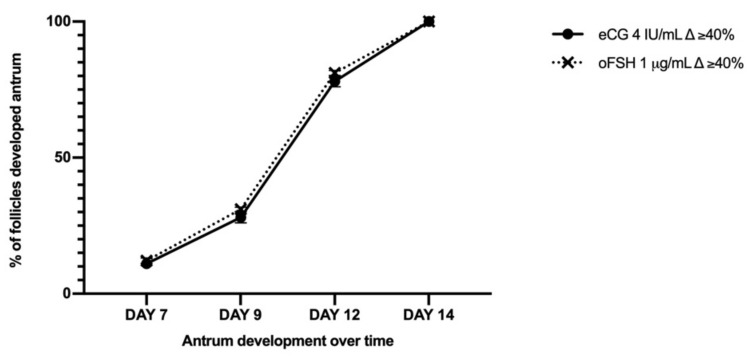
Development trend of the follicular antrum over time according to follicular treatment groups. Follicular development and antrum differentiation: Three independent biological replicates were performed. For each biological replicate, 66, 67, and 67 follicles per group (eCG 4 IU/mL and oFSH 1 μg/mL) were cultured.

The in vitro follicle culture carried out in an FBS-free medium lead to an overall improvement in follicle growth compared to that recorded in the FBS-supplemented medium. Indeed, the total of the EA follicles displayed a ∆ growth ≥40%, reaching a mean final diameter of 434 ± 31 and 413 ± 25 μm in the eCG and oFSH follicles, respectively. Any differences between the follicular parameters, such as the number of healthy follicles and the kinetics of antrum formation, were recorded in an FBS-free medium-*iv*F between the oFSH and eCG groups (Table 5 and Figure 4).

#### 2.3.2. Influence of eCG and oFSH on Oocytes Collected from ivF Carried Out in FBS Free Medium

**Figure 5:**
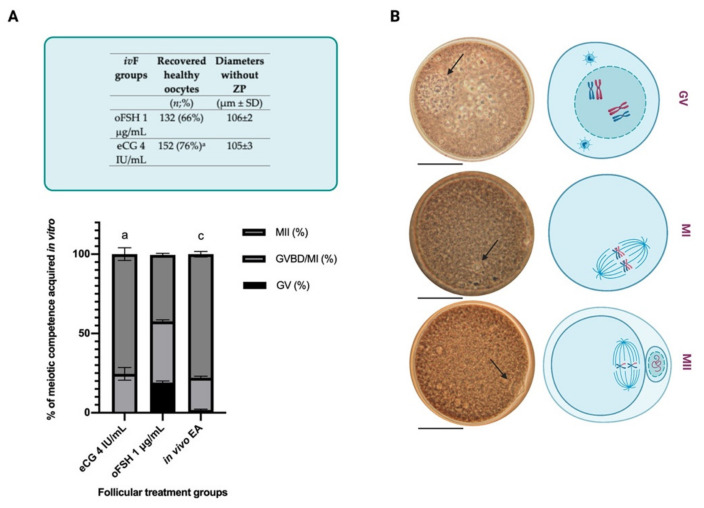
(**A**) Upper panel: table summarizing the number of recovered healthy oocytes and their mean diameters at the end of *iv*F culture. Letters indicate significant differences: ^a^ eCG 4 IU/mL vs. oFSH 1 μg/mL. Lower panel: percentage of the oocyte meiotic competence acquired in vitro. Different nuclear stages were characterized. Letters indicate significant differences between ^a^ eCG 4 IU vs. FSH 1μg/mL; ^c^ FSH 1μg/mL vs. in vivo EA. Three independent biological replicates were performed with 66, 67, and 67 follicles per group (eCG 4 IU/mL and oFSH 1 μg/mL). For each *iv*F group (oFSH 1 μg/mL and eCG 4 IU/mL) 132 and 152 follicles, respectively, were analyzed at the end of the in vitro culture for both follicular and oocyte performance. Only those follicles that differentiated the follicular antrum, identified on the stereomicroscope as a transparent cavity filled with follicular fluid, were tested. For each *iv*F group (oFSH 1 μg/mL and eCG 4 IU/mL), 132 and 152 healthy oocytes were recovered and analyzed at the end of the in vitro culture to test the oocyte performance. Only the oocytes retrieved from follicles that differentiated the follicular antrum, identified on the stereomicroscope as a transparent cavity filled with follicular fluid, were tested. (**B**) Images of oocyte nuclear stages. GV: Germinal Vesicle Break Down; MI: Metaphase 1; MII: Metaphase II (Lacmoid solution staining, 40× magnification; scale bar: 50 μm). Panel created with BioRender. Available online: https://biorender.com/ (accessed on 15 November 2021).

The highest percentage of healthy oocytes was collected from the EA follicles developed under *iv*F culture performed in an FBS-free medium (respectively, for eCG 76% and oFSH 66%: *p* < 0.0004; Figure 5). The assay of the nuclear stage showed a significantly higher % of MII oocytes in eCG (75.5%) than in oFSH (42%: see Figure 5). Notably, when the oocytes were exposed to eCG during *iv*F in FBS-free medium the acquisition of meiotic competence was similar to that recorded in the female gametes collected from the EA grown in vivo (75.5% vs. 77.7%, respectively). Specifically, no GV oocytes were found in the IVM oocytes isolated from the eCG-treated follicles vs. 19% of oFSH-derived follicles (see Figure 5).

### 2.4. Steroidogenic Transcript Program Activation on ivF Culture

**Figure 6:**
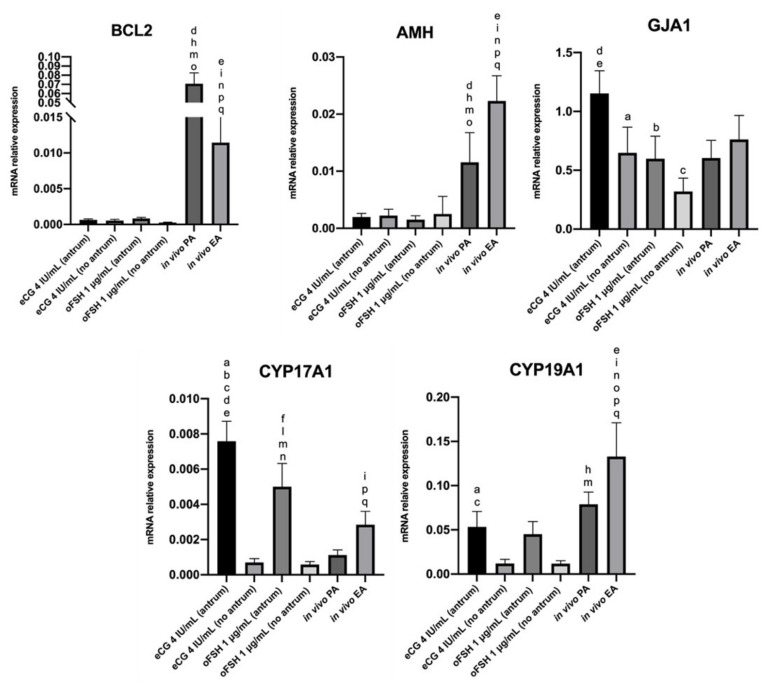
Representative expression of steroidogenic and somatic-specific genes. Letters indicate significant differences between ^a^ eCG 4 IU/mL (antrum) vs. eCG 4 IU/mL (no antrum); ^b^ eCG 4 IU/mL (antrum) vs. oFSH 1 μg/mL (antrum); ^c^ eCG 4 IU/mL (antrum) vs. oFSH μg/mL (no antrum); ^d^ eCG 4 IU/mL (antrum) vs. in vivo PA; ^e^ eCG 4 IU/mL (antrum) vs. in vivo EA; ^f^ eCG 4 IU/mL (no antrum) vs. oFSH 1 μg/mL (antrum); ^h^ eCG 4 IU/mL (no antrum) vs. in vivo PA; ^i^ eCG 4 IU/mL (no antrum) vs. in vivo EA; ^l^ oFSH 1 μg/mL (antrum) vs. oFSH 1 μg/mL (no antrum); ^m^ oFSH 1 μg/mL (antrum) vs. in vivo PA; ^n^ oFSH 1 μg/mL (antrum) vs. in vivo EA; ^o^ oFSH 1 μg/mL (no antrum) vs. in vivo PA; ^p^ oFSH 1 μg/mL (no antrum) vs. in vivo EA; ^q^ in vivo PA vs. in vivo EA. Three independent biological replicates were performed. For each biological replicate, 66, 67, and 67 follicles per group (eCG 4 IU/mL and oFSH 1 μg/mL) were cultured.

The steroidogenic transcript program activation (*CYP17A1* and *CYP19A1*) was assessed to evaluate the effect of the two gonadotropins (eCG vs. oFSH) and the coherence with the level of E2 secretion. Two representative genes whose expression is differentially regulated on in vitro developed follicle compared with in vivo follicles (*BCL2* and *AMH*), and *GJA1* as an indicator of the germinal–somatic coupling performance, were investigated to control of the different dynamics sustaining *iv*F growth and the acquisition of meiotic competence (Figure 6).

#### 2.4.1. Comparison In Vitro (eCG vs. oFSH, Antrum vs. No Antrum)

Focusing on the follicles grown in vitro, those treated with eCG showed a higher level of *GJA1* (2-fold change increase) compared with oFSH (eCG antrum vs. oFSH antrum). Notably, the antrum cavity formation was sustained by a marked expression of *GJA1*, as demonstrated by the expression of lower levels of *GJA1* in follicles without antrum cavity formation in eCG (1.8-fold change decrease) and oFSH (3-fold change decrease) conditions, respectively.

Moreover, the expression of steroidogenic enzymes (*CYP17A1* and *CYP19A1*) was consistent with the level of E2 secretion, showing a significant upregulation in the follicles treated with eCG 4 IU that developed antrum. A 10-fold change increase for *CYP17A1* and a 5-fold change increase for *CYP19A1* in eCG with antrum compared to eCG with no antrum were observed, respectively. Treatment with oFSH caused an 8-fold change increase in *CYP17A1* and a 4-fold increase in *CYP19A1* in the follicles that developed an antrum cavity when compared with those without antrum.

#### 2.4.2. Comparison In Vivo (PA vs. EA)

*AMH*, *GJA1*, *CYP17A1*, and *CYP19A1* gene targets were upregulated in in vivo grown EA follicles, whereas *BCL2* gene expression was predominantly represented in the PA follicles. Interestingly, when compared with the PA follicles, the EA in vivo follicles showed a 2.8-fold change increase inf *CYP17A1* and a 1.7-fold increase in *CYP19A1*.

#### 2.4.3. Comparison In Vitro vs. In Vivo

The in vivo PA follicles showed an upregulation of *BCL2* and *AMH* when compared with the follicles cultured in vitro. For what concerns steroidogenic enzymes, *CYP17A1* and *CYP19A1* showed different behavior in in vitro and in vivo contexts. Specifically, *CYP17A1* was predominantly expressed in in vitro cultured follicles, showing 2.5-fold change increase (eCG antrum vs. in vivo EA) and a 1.8-fold change increase (oFSH antrum vs. in vivo EA). Conversely, *CYP19A1* was predominantly expressed in in vivo EA follicles, showing a 2.6-fold change increase (in vivo EA vs. eCG antrum) and a 2.9-fold change increase (in vivo EA vs. eCG antrum).

### 2.5. Follicular Secretion of Estradiol (E2) during ivF Culture

**Figure 7:**
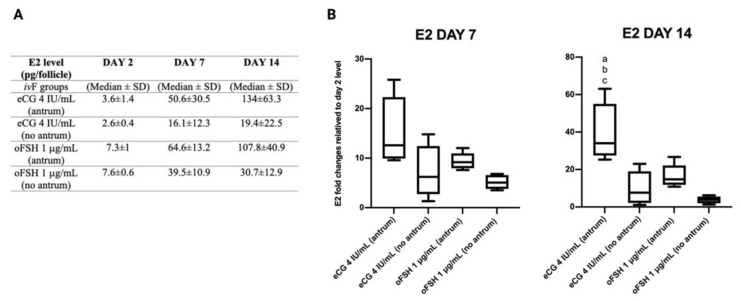
Table reporting median levels of E2 (pg/follicle) detected in conditioned follicular media in the different *iv*F culture conditions at Days 2, 7, and 14 (**A**). Data box-plot of estradiol concentrations in different follicular treatment groups during the culture period. Values of estradiol concentrations on day 7 and 14 were compared to the value on day 2 (first controlled point during the culture period) to evaluate dynamic of estradiol production. Letters indicate significant differences between ^a^ eCG 4 IU/mL (antrum) vs. eCG 4 IU/mL (no antrum); ^b^ eCG 4 IU/mL (antrum) vs. oFSH 1 μg/mL (antrum); ^c^ eCG 4 IU/mL (antrum) vs. oFSH μg/mL (no antrum) (**B**). Three independent biological replicates were performed. For each biological replicate, 66, 67, and 67 follicles per group (eCG 4 IU/mL and oFSH 1 μg/mL) were cultured and tested for this analysis.

The potential grade of follicular development in vitro was proved by testing the steroidogenic performance of the growing follicles in both gonadotropin treatments. Specifically, E2 secretion was evaluated during the *iv*F culture. The differential secretion of E2, which was dependent on both gonadotropin treatment and antrum formation, was evident as early as Day 7 of the *iv*F culture and became significant at Day 14 (Figure 7).

Indeed, eCG was more effective at inducing E2 secretion when compared to oFSH, and independently of gonadotropin treatment, the follicles that developed an antrum cavity produced a higher level of E2. At the end of the *iv*F culture, the effectiveness of eCG at enhancing steroidogenesis was significantly visible at Day 14, where the follicles that were treated with eCG and developed an antrum cavity produced significantly higher levels of estradiol compared to the other groups (See Figure 7). Specifically, E2 secretion was predominantly expressed in the eCG antrum, showing a 1.25-fold change increase (eCG antrum vs. oFSH antrum) and a 6.9-fold change increase (eCG antrum vs. eCG no antrum), while the oFSH antrum showed a 3.5-fold chance increase (oFSH antrum vs. oFSH no antrum).

**Figure 8:**
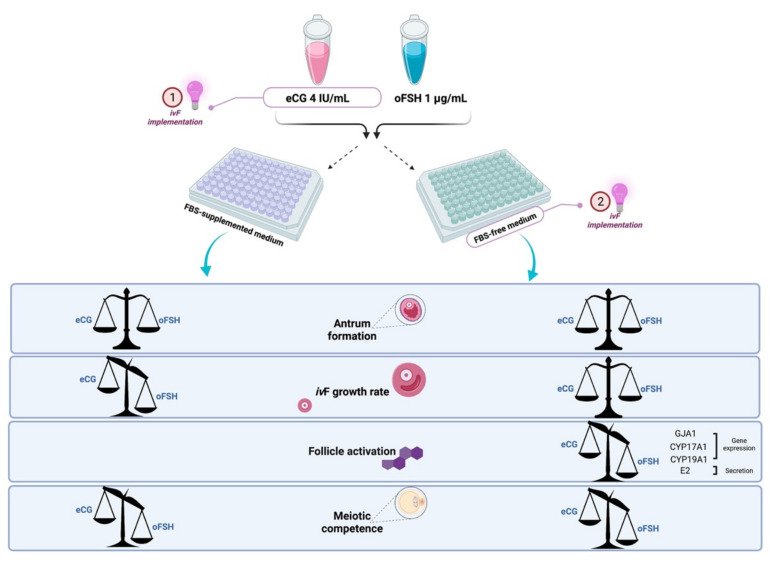
*iv*F outcome comparison between oFSH and eCG: *iv*F growth rate, antrum formation, follicle activation (steroidogenic transcriptional program activation and E2 secretion), and meiotic competence acquisition. Figure created with BioRender. Available online: https://biorender.com/ (accessed on 15 November 2021).

The gathered results are summarized in Figure 8.

## 3. Discussion

Over the past decades, follicle culture systems have been developed for promoting ovarian ivF in several mammalian species to reproduce in culture the growth of meiotic incompetent oocytes. Despite the high in vitro maturation rate achieved in current *iv*F protocols, the effect of some aspects, such as those related to epigenetic and environmental stimuli, on the oocyte quality need to be further investigated [59]. However, this advanced reproductive technique represents a potential strategy to improve animal reproduction/production and research trials, as well as to preserve human fertility. The biomedical interest in *iv*F has been further increased recently as a consequence of the increased rate of survival of young patients affected by cancer [60]. In this case, setting up validated *iv*F protocols represents a safe biotechnical strategy through which to restore fertility chances once patients reach adult life in a healthy condition instead of the transplantation of their ovarian tissues, which potentially exposes the organism to the risk of reintroducing malignant cells [61,62].

However, the ability to reproduce in vitro the first phases of folliculogenesis (follicle growth and oocyte development) still represents an unsolved challenge for reproductive biotechnology in mammals [17,23,63,64] as it requires wide margins of standardization, including the definition of the gonadotropic role [60].

FSH seems to exert an essential influence in vitro also during the phase preceding antrum cavity formation, when this hormonal stimulation has been considered essential to establish coordinated growth between the germinal and somatic compartments [47]. At the same time, FSH constrains *iv*F implementation due its high species specificity and its variable biological activity, which related to its source (recombinant or naïve). To overcome this limit, it might be of interest to study more handling solutions to promote *iv*F by substituting its stimulatory influence [65] or, alternatively, by adopting, as for animal superovulation, a chorionic gonadotropin with FSH-like activity.

A placental glycoprotein hormone, eCG is secreted by fetal trophoblastic epithelial cells constituting the endometrial cups [66]. It demonstrates biological activity similar to FSH, physiologically causing follicles ovulation and leading to produce accessory corpora lutea which helps to support the developing fetus [67].

For a long time, eCG was used as an alternative FSH hormone for superovulation induction by improving the process of follicle recruitment in several mammal models [68]. Indeed, it was successfully used in small mammals, such as mice rats [69] rats alone [70], medium-large sized mammals such as sheep [71,72,73], goats [74], cows [75], mares [76], and pigs [77].

Notably, its use has been consolidated over recent years due to its longer circulatory half-life, enhanced trans-species effect, lower cost, and greater commercial availability compared with FSH [54,55]. Despite these numerous advantages, no evidence has been collected to date documenting eCG’s influence on the promotion of in vitro folliculogenesis.

Based on our results, 4 IU/mL eCG was found to efficiently support proper ovine PA follicle growth by inducing the synchronous development of germinal and somatic compartments. Furthermore, eCG appears much more effective in improving some key cultural biological targets of *iv*F, such as follicle growth, estradiol synthesis, and the acquisition of oocyte meiotic competence. Overall, the evidence presented in this paper suggests widespread eCG use as a substitute for oFSH in *iv*F protocols.

In more detail, while eCG and FSH demonstrated a similar biological influences on the percentage of antrum formation, healthy follicles, and recovered oocytes, conversely, chorionic gonadotropin supplementation showed the ability to significantly enhance the incidence of follicles with greater rates of growth (Δ growth). Indeed, almost all the eCG-treated follicles (81%) displayed a Δ growth greater than that of the average PA follicles’ Δ growth (>40%). The achievement of such of growth rates positively influenced the oocytes’ quality.

Indeed, oocytes expressing the highest IVM meiotic competence (68% MII in eCG vs. 43% MII with oFSH) were isolated from precisely this category of EA follicles (PA follicles with a rate of growth > of medium Δ growth). This effect was observed either when *iv*F was carried out in the FBS-supplemented medium or under improved *iv*F conditions, such as those performed using the FBS-free medium on. Notably, by coupling the use of eCG with the beneficial effect of FBS-free medium supplementation, the performance of meiotic competence acquisition was similar to that of the oocytes collected from the in vivo EA follicles (75.5% vs. 77.7% MII oocytes, respectively). Accordingly, Park et al. demonstrated how the replacement of FBS-supplemented medium with an FBS-free medium exerted a useful effect on oocyte maturation systems, contributing to identifying the functional elements necessary for folliculogenesis [58]. Notably, the use of FBS-free medium supported higher follicular survival rates when compared to the medium supplemented with FBS [56].

In our study, the FBS-free medium was able to positively affect follicle development by equalizing the timing of antrum formation and shortening the window period needed for the antrum cavity development regardless of whether the treatment was eCG or oFSH. In addition, all the follicles that developed an antrum cavity in FBS-free medium showed a Δ growth of more than 40% (for eCG: 76% and for oFSH: 66%).

This effect may have been the result of a longer in vitro persistence or a greater degree of metabolic coupling between the somatic and germinal compartments under reduced concentrations of FBS [17,64]. Any information on oocyte–follicle coupling were collected in the present research; however, indirect evidence may confirm this pro-healthy effect of FBS removal. Indeed, focusing attention on the in vitro grown follicles developing antrum cavity, those treated with eCG in combination with FBS free-medium, showed a marked expression of GJA1. The greater expression of GJA1 may be indicative of better support in the synchronization of the process involving the proliferation of granulosa cells, indirectly suggesting that treatment with eCG guarantees an efficient coupling of the somatic and germinal compartments, which in turn reflects the observed meiotic competence acquisition performance. A lower expression in those follicles that did not develop an antrum cavity during e *iv*F could be justified as a lack of communication between oocyte and granulosa cells. Indeed, the PA follicles collected after 14 days of culture in the FBS-supplemented medium showed a higher incidence of degenerated follicles (approximately 40% in *iv*F FBS-supplemented medium vs. 34% and 24% in follicles treated with oFSH and eCG in FBS-free medium) as well as a smaller percentage of EA (for oFSH and eCG respectively: 58% and 61% in FBS-supplemented medium vs. 66% and 76% in the FBS-free medium). Moreover, it was possible to notice that the antrum cavity differentiation recorded in the follicles treated with eCG and cultured in the FBS-free medium led to the in vitro achievement of a high degree of follicular maturity, as evidenced by the steroidogenic gene expression profiles and the estradiol release in the culture medium.

More specifically, the superior in vitro performance of eCG in sustaining the follicular wellness in combination with the FBS-free medium was confirmed by the activation of the transcriptional steroidogenic program and by the secretion of E2. Notably, the steroidogenic enzymes *CYP17A1* and *CYP19A1* were upregulated in those follicles developing antrum cavity in both treatments. However, eCG was able to activate the steroidogenic transcriptional program with higher efficiency, as demonstrated by the more evident and significant expression of steroidogenic enzymes in the follicles that developed antrum cavity compared to that recorded in the oFSH.

The superior performance of eCG may have been due to its LH-like action, as demonstrated by the recorded upregulation of the theca-specific *CYP17A1* gene. Moreover, our data clearly suggest that antrum development represents a key factor in the modulation of *CYP17A1* expression, since only the follicles with an antrum cavity showed a significant upregulation of this gene. Consistently, *CYP19A1* upregulation could be interpreted as a result of a boosted production of precursors for estrogen synthesis as well as a crosstalk between oocyte, granulosa, and theca cells, representing a key element of follicular well-being. Importantly, E2 secretion in the conditioned culture media showed a coherent trend of accumulation. The greater functional maturation of the follicles that developed an antrum cavity in the presence of eCG in the FBS-free medium also positively reflected improved oocyte development, especially in terms of the acquisition of meiotic competence.

In light of this evidence and previous studies that p documented a correlation between antrum cavity formation and follicular maturation degree [17,31,64,78,79,80] the use of antrum cavity formation as a biological marker of successful follicular growth could be hypothesized.

Moreover, the synergistic FSH and LH-like activity of eCG, was documented to represent a key element of proper follicle growth, ovulation, and corpus luteum formation [81,82].

**Figure 9:**
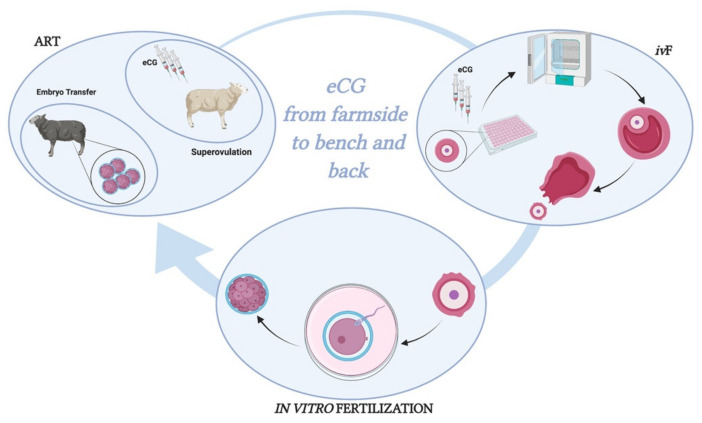
eCG: from farmside to bench and back. Figure created with BioRender. Available online: https://biorender.com/ (accessed on 15 November 2021).

The stimulatory effect of eCG was validated by verifying the ability of chorionic gonadotropin to improve the outcomes of the in vitro follicular development, increasing the in vitro follicle growth rate % and enhancing the gene expression of steroidogenic enzymes and estradiol releasing. Similarly, eCG promoted synchronous oocyte growth and follicle development, as demonstrated by its ability to induce an efficient meiotic resumption, reaching IVM outcomes similar to those recorded in the in vitro maturated oocytes derived from the in vivo grown EA follicles. Many other advantages resulting from the use of eCG to support in vitro follicle culture should also be considered: its commercial availability, longer circulatory half-life, trans-species action, and lower costs. Moreover, chorionic gonadotropin can extend its biological power to in vitro cultural systems according to its FSH-like activity, as successfully demonstrated in validated superovulation protocols (Figure 9).

## 4. Materials and Methods

### 4.1. Chemicals

All the chemicals used in this study were purchased from Sigma (Sigma Chemical Co., St. Louis, MO, USA), unless otherwise indicated.

### 4.2. Isolation, Morphological Evaluation, and In Vitro Culture of PA Follicles

The present research was carried out by collecting *Appenninica* sheep breed lamb ovaries (about 5 months old) from discarded tissues, picked from animals intended for consumption, at a slaughterhouse [78].

From the ovaries from prepubertal sheep were collected from the local slaughterhouse and transported to the laboratory in a thermostatic container within 1 h of collection. The ovaries were then rinsed several times in NaCl 0,9% solution supplemented with Benzoxonium chloride 1 mg/mL (Bialcol Med, Vemedia Pharma S.r.l., Parma, Italy). The ovaries were kept at 38 °C during transportation from the slaughterhouse to the laboratory.

After medulla removal, the ovaries were transferred into an HEPES-buffered TCM199 medium (RNBG9152 Sigma) and cut into cortical fragments (0.5 × 0.5 × 0.5 cm). The PA follicles were mechanically isolated from the cortical fragments thanks to 32 G sterile needles under ae stereomicroscope in the flow hood and selected on the basis of their morphology and size [23] in order to keep the theca layer intact. The large PA follicles used for in vitro culture experiments displayed a mean diameter of approximal 250 μm. The PA follicles were accurately measured under an inverted-phase microscope associated with the time-lapse imaging software, NIS-Elements (Eclipse Ti Series, Nikon Europe BV, Amsterdam, Netherlands), in order to exclude follicles with damaged basal membrane, discard early antral (EA) follicles, and select PA follicles with good in vitro performance [20,23,64]. The in vitro grown PA follicles displayed a final mean diameter of approximal 386 μm after 14 days of in vitro culture.

### 4.3. In Vitro Culture of PA Follicles

Isolated PA follicles with a morphological appearance that excluded signs of early degeneration (i.e., darkness of follicles, non-round shape) were recorded, randomly assigned to one of the experimental groups, and set up for 14 days in single-follicle in vitro culture, as described by Barboni et al. [17].

The follicles were incubated as 3D single follicles, placed in a 25 μL drop of culture medium under a 75 μL drop of pre-equilibrated mineral oil (density 5 0.84 g/mL) and then overlaid by 25 μL oil in a 96 well dish with V-shaped wells (Nunc, Roskilde, Denmark).

In order to compare the effect of eCG and oFSH on the ovine PA follicle culture, a dual cultural approach was adopted and simultaneously tested: (1) *iv*F was performed in an FBS-supplemented medium, as previously reported [17,64]. This was achieved through MEM alpha modification (aMEM; 8MB170 Lonza) supplemented with 2% fetal bovine serum (FBS), 1% ITS (insulin, transferrin, and selenium; I1884 Sigma), 50 μg/mL ascorbic acid (SLCC2424 Sigma), 2 mM glutamine, and antibiotics (75 mg/L penicillin-G, 50 mg/L streptomycin sulfate). (2) *iv*F was performed in an FBS-free medium: MEM alpha (aMEM; 8MB170 Lonza) with 5% Knockout™ Serum Replacement (Knockout™ SR; 10828 Gibco), 1% ITS (insulin, transferrin, and selenium; I1884 Sigma), 50 μg/mL ascorbic acid (SLCC2424 Sigma), 2 mM glutamine, and antibiotics (75 mg/L penicillin-G, 50 mg/L streptomycin sulfate).

The FSH treatment group was designed by in vitro stimulation of the PA follicles with 1 μg/mL FSH from ovine pituitary/oFSH (corresponding to 25 mIU/mL; F8174 Sigma) as previously set up [17,64,78]. The biological activity of the oFSH was declared to be 50 IU per vial, according to the Steelman–Pohley Assay using the NIH-FSH-S1 reference standard [82]. The eCG treatment group was designed by in vitro stimulation of the PA follicles with 0.4 μg/mL eCG (corresponding to 4 IU/mL; FOLLIGON^®^, MSD Animal Health S.r.l., Milan, Italy). The biological activity of the eCG was declared to be 5000 IU per vial, according to the manufacturer’s instructions. The eCG 4 IU/mL dose was selected after designing a dose-curve experiment testing the following point concentrations: eCG 0.4, 4, and 40 IU/mL (corresponding approximatively to 0.1, 1 and 8 µg/mL, respectively).

The culture was carried out at 38.5 °C and 5% CO_2_ for 14 days. The culture medium (25 μL) was changed every 48 h and the conditioned media were stored at −80 °C for further steroid determination.

### 4.4. Experimental Plan

To determine the optimal chorionic gonadotropin concentration capable of stimulating PA follicle growth in vitro, a preliminary dose effect on large PA follicle culture was set up. The follicles (360 in total) were divided into 3 groups consisting of 90 PA follicles each, which were treated with an increasing dose of eCG (0, 0.4, 4, and 40 IU/mL) and compared with the oocytes derived from in vivo early antral (EA) follicles (mean diameter: 353 ± 8 μm) for the acquisition of meiotic competence.

#### 4.4.1. Comparison between eCG vs. oFSH

The experiments aimed to study the influence of two different gonadotropins (oFSH vs. eCG) on the in vitro follicle culture (*iv*F) of a single-ovine large PA follicle incubated contextually in FBS-supplemented and FBS-free media. The *iv*F outcomes were analyzed after 14 days of culture by comparing the follicle/oocyte growth, timing, and percentage (%) of antrum differentiation, as well as the percentage of Metaphase II (MII) oocytes after in vitro Maturation (IVM) and estradiol determination by Estradiol EIA kit (501890; Cayman Chemical Company, Ann Arbor, MI, USA).

#### 4.4.2. IvF Outcomes

##### Morphological Analysis of In Vitro Follicle Development

By the end of the culture, the final follicle diameters were recorded, together with the presence or absence of an antral cavity, which was defined as a visible translucent area within the granulosa cell mass comprising about half of the follicle. The follicles were scored for their morphological appearance, and those showing morphological signs of degeneration (i.e., darkness of oocytes and surrounding cumulus cells), or those with misshapen oocytes were discarded. Furthermore, the follicle selection after the in vitro culture was performed considering some morphological criteria in order to distinguish healthy follicles from unhealthy follicles (degenerated and no-antrum follicles).

Healthy follicles were defined by considering follicular integrity, the absence of visible signs of degeneration (darkness of the oocyte and follicular cells) and the absence of extrusion of the oocyte. The formation of a cavity (follicles with antrum) was identified under the stereomicroscope and confirmed using an inverted microscope (time-lapse imaging software, NIS-Elements (Eclipse Ti Series, Nikon, Japan), through the presence of a translucent cavity inside the in vitro grown follicles. The unhealthy follicles category was characterized by: (1) follicles with no visible sign of degeneration and without antrum cavity formation, named as “no-antrum” follicles; (2) follicles without follicular integrity, presenting extrusion of the oocytes, and that did not display antrum cavity development, named as “degenerated” follicles.

The final follicle and oocyte diameters, as well as antrum formation and, eventually, signs of degeneration, were measured using the time-lapse imaging software, NIS-Elements (Eclipse Ti Series, Nikon Europe BV, Amsterdam, Netherlands)

#### 4.4.3. Oocytes IVM of In Vitro Grown (IVG) Follicles

At the end of the culture and according to follicular growth ∆% (≥ or <40%), cumulus-oocyte complexes (COCs), derived from IVG early antral follicles, were mechanically isolated from the follicles with 32 G sterile needles under the stereomicroscope under the flow hood.

COCs presenting continuous and compact layers of cumulus cells and corresponding oocytes without signs of cytoplasmic degeneration were classified as healthy. The diameters of oocytes obtained from IVG follicles were also recorded.

The COCs were divided into a 4 well plate (Nunc, Roskilde, Denmark) according to the experimental group (increasing doses of eCG (0, 0.4, 4, and 40 IU/mL) vs. oocytes derived from in vivo EA follicles, 1 μg/mL oFSH vs. 4 IU/mL eCG in (1) FBS-supplemented and (2) FBS-free mediums and co-cultured with a monolayer of ovarian surface epithelium (OSE) cells derived from adult sheep ovaries, through an advanced IVM method, and left in a maturation culture medium (alphaMEM, 20% fetal bovine serum (FBS), 1% Ultraglutammine, antibiotics like 75 mg/L penicillin-G and 50 mg/L streptomycin sulfate, hCG and eCG 10 IU/Petri) in an incubator for 24 h prior to the assay.

#### 4.4.4. Oocyte Meiotic Competency Assessment

The meiotic competency of the oocytes was compared between the groups (increasing doses of eCG (0, 0.4, 4 and 40 IU/mL) vs. oocytes derived from in vivo EA follicles, 1 μg/mL oFSH vs. 4 IU/mL eCG in (1) FBS-supplemented and (2) FBS-free mediums after in vitro maturation (IVM) of COCs derived from the early antral follicles obtained in vitro. After oocyte fixation, the Metaphase II (MII) stage was detected by staining the oocytes with 1% Lacmoid [17]. Next, the oocytes were mounted on an object slide and analyzed under a Phase Contrast Microscope (AxioVert, Carl Zeiss, Oberkochen, Germany).

#### 4.4.5. Detection of Estradiol in the Culture Medium (Estradiol Production Assay)

The estradiol-17b levels released by the follicles during the 14 days of in vitro culture were assessed using an Estradiol EIA kit (501890; Cayman Chemical Company, Ann Arbor, MI, USA).

The conditioned media (25 μL) used for this analysis were retrieved at Days 2, 7, and 14 of the in vitro follicle cultures, considering that the media replacement was performed every 48 h. The sensitivity of the assay, defined as the amount of steroid giving a 10% drop with binding of the enzyme-conjugated estradiol, was 6 pg/mL. The intra and interassay precision, expressed as the coefficients of variations for replicate determination of the sample, were 3.2% (10 replicates) and 4.9% (10 replicates), respectively. The levels of estradiol in the samples of conditioned media were expressed as pg/follicle.

**Figure 10:**
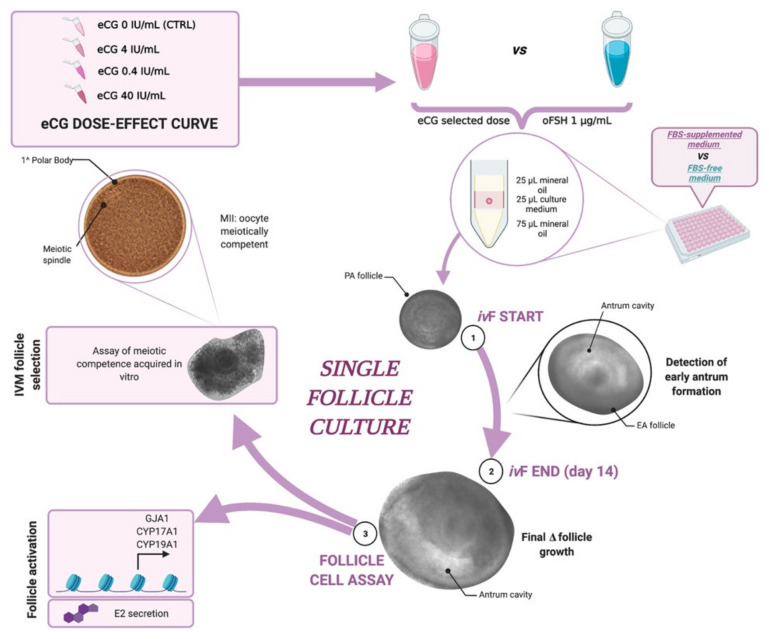
Flow chart of the experimental procedure adopted for culturing PA follicles in vitro. Figure created with BioRender. Available online: https://biorender.com/ (accessed on 15 November 2021).

The overall flow chart of the experimental plan is reported in Figure 10.

#### 4.4.6. Real-Time qPCR

The total RNA was extracted with a Single-Cell RNA Purification Kit (Norgen Biotek Corp. Cat 51800) following the manufacturer’s instructions. A total of 1 μg of total RNA was retrotranscribed using oligodT primers (Bioline, London, UK) and Tetro Reverse Transcriptase (Bioline, London, UK), following the manufacturer’s instructions. The qPCRs were carried out in triplicate using the SensiFAST SYBR Lo-ROX kit (Bioline London, UK) on a 7500 Fast Real-Time PCR System (Life Technologies, Carlsbad, CA, USA), according to the manufacturer’s instructions. The following PCR conditions were used for all the experiments: 95 °C for 10 min, followed by 40 cycles at 95 °C for 10 s and 60 °C for 30 s. Relative quantification was performed by using the ∆∆Ct method. *GAPDH* (Glyceraldehyde 3-phosphate dehydrogenase) was selected amongst the housekeeping genes for gene quantification. The expression profiles were similar with both reference genes. The primer sequences are reported in Table 6.

### 4.5. Statistical Analysis

Three independent biological replicates were performed. The data are presented as the percentage or mean ± SD. GraphPad Prism 9 (GraphPad Software) was used for the statistical analyses, and values with *p* < 0.05 were considered statistically different.

Differences in antrum formation, the percentage of degenerated follicles, and the achievement of oocyte meiotic competence in vitro between different treatments were evaluated by ordinary one-way ANOVA followed by the Tukey–Kramer test for the comparison of multiple groups. All the other data were analyzed through an unpaired *t*-test.

## Figures and Tables

**Table 1:** eCG dose effect influence on *iv*F outcomes: quantitative and qualitative PA follicle performances.

*iv*F Groups	*N°* of *iv*F PA	Unhealthy Follicles	Recovered Healthy Follicles	Degree of PA Growth	PA Diameter	EA Diameter
	(*n*)	(%)	(*n*;%)	(Δ%)	(μm ± SD)	(μm ± SD)
eCG 40 IU/mL	90	60 (67%) ^d^	30 (33%) ^d^	38%	243.8 ± 11	337 ± 29 ^a^
eCG 4 IU/mL	90	36 (40%) ^b,d,f^	54 (60%) ^b,d,f^	41%	257.6 ± 23	362 ± 50
eCG 0.4 IU/mL	90	59 (66%) ^f^	31 (34%) ^f^	46%	252.3 ± 19	364.2 ± 44
eCG 0 IU/mL (CTRL)	90	61 (68%) ^a,c,f^	29 (32%) ^a,c,f^	10%	233.2 ± 3	256.2 ± 2 ^a^

Letters indicate significant differences: ^a^ 0 vs. eCG 40 IU/mL; ^b^ 0 vs. eCG 4 IU/mL; ^c^ 0 vs. eCG 0.4 IU/mL; ^d^ eCG 40 IU/mL vs. eCG 4 IU; ^f^ eCG 4 IU/mL vs. eCG 0.4 IU/mL. Three independent biological replicates were performed. For each biological replicate, 30 follicles per group (eCG 40, 4, 0.4, 0 IU/mL) were cultured; for each *iv*F group (eCG 40, 4, 0.4 and 0 IU/mL (CTRL) 30, 54, 31, and 29 follicles, respectively, were analyzed at the end of the in vitro culture for both follicular and oocyte performance. Only healthy follicles that differentiated the follicular antrum, identified on the stereomicroscope as a transparent cavity filled with follicular fluid, were tested. Unhealthy follicles referred to degenerated and no-antrum follicles. Parameters such as degree of PA follicle growth and % of follicles developing antrum over time were calculated from healthy follicles (follicles with antrum cavity formation).

**Table 2:** eCG dose-effect on oocyte growth and meiotic competence.

*iv*F Groups	*N°* of *iv*F PA	Recovered Healthy Oocytes	Oocyte Diameters without ZP	GV	GVBD/MI	MII
	(*n*)	(*n*;%)	(μm ± SD)	(%)	(%)	(%)
eCG 40 IU	90	30 (33%)	103 ± 5 ^a^	69.3% ^d,e^	10%	20.7%
eCG 4 IU	90	54 (60%) ^i^	106 ± 4 ^b^	5.6%	27.7% ^b,d,f^	66.7% ^b,e,h,i^
eCG 0.4 IU	90	31 (34%)	105 ± 6 ^c^	42%	10%	48% ^c,f^
eCG 0 IU (CTRL–)	90	29 (32%)	93 ± 2	71% ^a,b,c^	7%	22%
in vivo EA (CTRL+)	90	80 (89%) ^i^	106 ± 3 ^d^	1.7%	20.6% ^d,g,l^	77.7% ^d,g,l^

Letters indicate significant differences: ^a^ 0 vs. eCG 40 IU; ^b^ 0 vs. eCG 4 IU; ^c^ 0 vs. eCG 0.4 IU; ^d^ 0 vs. in vivo EA; ^e^ eCG 40 IU vs. eCG 4 IU; ^f^ eCG 40 IU vs. eCG 0.4 IU; ^g^ eCG 40 IU vs. in vivo EA; ^h^ eCG 4 IU vs. eCG 0.4 IU; ^i^ eCG 4 IU vs. in vivo EA; ^l^ eCG 0.4 IU vs. in vivo EA. Three independent biological replicates were performed with 30 follicles per group (eCG 40, 4, 0.4, 0 IU/mL). For each *iv*F group (eCG 40, 4, 0.4, 0 IU/mL/CTRL− and in vivo EA/CTRL+) 30, 54, 31, 29, and 80 healthy oocytes were recovered and analyzed at the end of the in vitro culture to test the oocyte performance. Only the oocytes retrieved from follicles that differentiated the follicular antrum, identified on the stereomicroscope as a transparent cavity filled with follicular fluid, were tested.

**Table 3:** eCG and oFSH effect on *iv*F outcomes: quantitative and qualitative PA follicle performances in FBS-supplemented medium.

*iv*F Groups	*N°* of *iv*F PA	Recovered Healthy Follicles	∆ ≥ 40%	PA Diameter	EA Diameter
	(*n*)	(*n*;%)	(*n*;%)	(μm ± SD)	(μm ± SD)
oFSH 1 μg/mL	200	116 (58%)	60%	254 ± 26	380 ± 70
eCG 4 IU/mL	200	122 (61%)	81% ^a^	256 ± 25	410 ± 68

Letters indicate significant differences: ^a^ eCG 4 IU/mL ∆ ≥ 40% vs. oFSH 1 μg/mL ∆ ≥ 40%. Three independent biological replicates were performed with 66, 67, and 67 follicles per group (eCG 4 IU/mL and oFSH 1 μg/mL). For each *iv*F group (oFSH 1 μg/mL and eCG 4 IU/mL) 116 and 122 follicles, respectively, were analyzed at the end of the in vitro culture for both follicular and oocyte performance. Only those follicles that differentiated the follicular antrum, identified on the stereomicroscope as a transparent cavity filled with follicular fluid, were tested. For each *iv*F group (oFSH 1 μg/mL and eCG 4 IU/mL), 116 and 122 healthy oocytes were recovered and analyzed at the end of the in vitro culture to test the oocyte performance. Only the oocytes retrieved from follicles that differentiated the follicular antrum, identified on the stereomicroscope as a transparent cavity filled with follicular fluid, were tested. Parameters such as degree of PA follicle growth and % of follicles developing antrum overtime were calculated from healthy follicles (follicles with antrum cavity formation).

**Table 4:** Comparison of eCG and oFSH effect on growth and meiotic competence of oocytes isolated from *iv*F PA in FBS-supplemented medium.

*iv*F Groups	Recovered Healthy Oocytes	Diameters without ZP
	(*n*;%)	(μm ± SD)
eCG 4 IU/mL (αω)	122 (61%)	107 ± 5
∆ > 40%	99 (81%) ^a^	
∆ < 40%	23 (19%) ^f^	
oFSH 1 μg/mL (αω)	116 (58%)	105 ± 7
∆ > 40%	70 (60%)	
∆ < 40%	46 (40%)	

Letters indicate significant differences: ^a^ eCG 4 IU ∆ ≥ 40% vs. FSH 1 ug/mL ∆ ≥ 40%; ^f^ eCG 4 IU ∆ < 40% vs. FSH 1 μg/mL ∆ < 40%. Three independent biological replicates were performed with 66, 67, and 67 follicles per group (eCG 4 IU/mL and oFSH 1 μg/mL). The value (αω) indicates all the oocytes recovered by follicular treatments independently of their ∆ growth%.

**Table 5:** Gonadotropin influence on *iv*F outcomes: quantitative and qualitative aspects according to follicular treatment groups.

*iv*F Groups	*N°* of *iv*F PA	Recovered Healthy Follicles	Degree of PA Growth	PA Starting Diameter	Final Diameter
	(*n*)	(*n*;%)	(∆ > 40%)	(μm ± SD)	(μm ± SD)
oFSH 1 μg/mL	200	132 (66%)	68.7%	254 ± 17	413 ± 25
eCG 4 IU/mL	200	152 (76%) ^a^	70%	256 ± 15	434 ± 31

Letters indicate significant differences: ^a^ eCG 4 IU/mL vs. oFSH 1 μg/mL. Three independent biological replicates were performed with 66, 67, and 67 follicles per group (eCG 4 IU/mL and oFSH 1 μg/mL). For each *iv*F group (oFSH 1 μg/mL and eCG 4 IU/mL) 132 and 152 follicles, respectively, were analyzed at the end of the in vitro culture for both follicular and oocyte performance. Only those follicles that differentiated the follicular antrum, identified on the stereomicroscope as a transparent cavity filled with follicular fluid, were tested. For each *iv*F group (oFSH 1 μg/mL and eCG 4 IU/mL), 132 and 152 healthy oocytes were recovered and analyzed at the end of the in vitro culture to test the oocyte performance. Only the oocytes retrieved from follicles that differentiated the follicular antrum, identified on the stereomicroscope as a transparent cavity filled with follicular fluid, were tested. Parameters such as degree of PA follicle growth and percentage of follicles developing antrum overtime were calculated from healthy follicles (follicles with antrum cavity formation).

**Table 6:** Sequences of primers used in real-time qPCR.

Gene	Forward Sequence	Reverse Sequence
*BCL2*	5′-CCTTCTTTGAGTTCGGAG-3′	5′-CTGGCTGTCTCTGAAGG-3′
*AMH*	5′-GTGGTGCTGCTGCTAAAGATG-3′	5′-CTCCTCATCAGCCTGTCCGA-3′
*GJA1*	5′-CACTTGAAGCAGATTGAA-3′	5′-AGGATACTGATGATGTAGG-3′
*CYP17A1*	5′-CTTACCATTGACAAAGGCACAGAC-3′	5′-CAACTCATCTCGCCATCATTAAGC-3′
*CYP19A1*	5′-TCGTCCTGGTCAACCCTTCTG-3′	5′-CCAGACGAGACCAGAGACCG-3′
*GAPDH*	5′-TCGGAGTGAACGGATTTGGC-3′	5′-CCGTTCTCTGCCTTGACTGT-3′
